# Risk Factors for Retinopathy of Prematurity in Very-Low-Birth-Weight Preterm Infants, With Special Reference to Postnatal Weight Gain

**DOI:** 10.7759/cureus.114145

**Published:** 2026-08-07

**Authors:** Sweta Mishra, Geetanjali Sethy, Lipika Nayak, Swarupa Panda

**Affiliations:** 1 Department of Pediatrics, Sriramachandra Bhanja Medical College and Hospital, Cuttack, IND

**Keywords:** india, insulin-like growth factor-1 (igf-1), neonatal screening., premature infants, retinopathy of prematurity (rop)

## Abstract

Objective: This study aimed to evaluate the relationship between postnatal weight gain and the risk of developing severe retinopathy of prematurity (ROP) in preterm neonates with very-low-birth-weight (VLBW) and extremely low birth weight (ELBW).

Methodology: This prospective observational study was conducted in the Special Neonatal Care Unit (SNCU) of Sriramachandra Bhanja Medical College and Hospital (SCBMCH) and Sardar Vallabh Bhai Patel Postgraduate Institute of Paediatrics (SVPPGIP), Cuttack, from January 2023 to July 2024. Preterm neonates with birth weight < 1,500 g and gestational age < 34 weeks were enrolled, excluding those with significant congenital or ocular anomalies or requiring ventilator support. Detailed clinical history, risk factors, and serial weight measurements were recorded until six weeks postnatal age. Data were analyzed using SPSS (IBM Corp., Armonk, NY, USA), employing univariate analyses, chi-squared tests, Mann-Whitney tests, and logistic regression to identify risk factors for ROP.

Results: Of 200 neonates, severe ROP was observed in 124 (38%). Logistic regression identified lower gestational age (odds ratio (OR) = 81.639), lower birth weight (OR = 11.371), apnea (OR = 34.594), and poor weight gain during the second and third weeks as significant risk factors for severe ROP. Mean weight gain increased from 8.3 ± 3.4 g/kg/day in the second week to 16.2 ± 5.4 g/kg/day by the sixth week. Poor weight gain in the second (OR = 0.699) and third (OR = 0.556) weeks was associated with a higher risk of severe ROP.

Conclusion: While low gestational age and birth weight remain primary risk factors, additional factors, including prolonged oxygen therapy, bronchopulmonary dysplasia, anemia, and repeated blood transfusions, also contribute to ROP severity. Infants with poor weight gain in the second and third weeks post-birth had a significantly higher risk of developing severe ROP (p < 0.0001), highlighting the importance of early nutritional monitoring in high-risk preterm neonates.

## Introduction

Retinopathy of prematurity (ROP) is characterized by abnormal retinal blood vessel growth in premature infants, which can lead to retinal detachment and blindness. The rising incidence of preterm births over the past decade has made ROP a leading cause of preventable childhood blindness. In India, the country is currently experiencing its “third wave” of ROP: the first wave was driven by unregulated oxygen therapy, the second by increased survival of very preterm infants, and the third is attributed to factors such as inadequate neonatal care and limited access to ROP screening and treatment [1-3].

ROP develops in two stages [4]. Retinal vascularization begins around the 16th week of gestation, spreading from the optic disc and typically completing by 36-40 weeks. In full-term infants, this process is complete, protecting ROP. Preterm infants, however, have underdeveloped retinas, with the degree of development dependent on gestational age (GA). The first stage, termed the vascular attenuation phase, is characterized by insufficient retinal vessel growth, leading to hypoxia. The second stage, the fibroproliferative phase, begins around 32-34 weeks postmenstrual age (PMA), when vascular growth factors are released [5-7]. Both oxygen-related and non-oxygen-related factors drive abnormal neovascularization during this stage. Vascular endothelial growth factor (VEGF) is oxygen-regulated, while insulin-like growth factor-1 (IGF-1) is a non-oxygen-regulated factor [8]. Low IGF-1 levels impair normal vessel development, whereas higher levels promote neovascularization.

Premature infants with ROP frequently exhibit low IGF-1 levels [9], which are closely associated with poor postnatal weight gain. Reduced weight gain limits IGF-1-mediated VEGF activation in the retina, impairing vascular development and significantly increasing the risk of severe ROP. Consequently, postnatal weight gain serves as an indirect marker of serum IGF-1 levels. Understanding the interplay between low IGF-1, insufficient weight gain, and ROP is critical for implementing early intervention and preventive strategies [6,9-11].

In India, the Rashtriya Bal Suraksha Karyakram (RBSK) and National Neonatology Forum (NNF) guidelines (2019-20) recommend screening for ROP in infants with a birth weight (BW) < 2,000 g (or <1,750 g per NNF) or a GA < 34 weeks. Infants with a GA of 34-36 weeks are screened if additional risk factors are present, including respiratory problems, prolonged oxygen therapy, sepsis, or intraventricular hemorrhage (IVH). Infants with unstable clinical courses, as assessed by a neonatologist or pediatrician, are also considered high-risk [12]. Initial screening is generally performed at four weeks of age, except for infants < 28 weeks of gestation or BW < 1,200 g, who are screened at 2-3 weeks. PMA is calculated by adding GA to chronological age. Incorporating postnatal weight monitoring into the screening process enables clinicians to identify infants requiring earlier or more frequent examinations and to prioritize high-risk infants, particularly in resource-limited settings, thereby improving timely intervention and outcomes.

The primary aim of this study was to identify potential risk factors associated with the development of ROP in preterm and very-low-BW (VLBW) infants. The study also aimed to assess the pattern of postnatal weight gain in these neonates and to determine the relationship between postnatal weight gain and the occurrence of severe ROP. By evaluating these parameters, the study sought to provide insights into early identification and management strategies for infants at high risk of developing ROP.

## Materials and methods

Study design and setting

This prospective observational study was conducted in the Special Neonatal Care Unit (SNCU) of Sriramachandra Bhanja Medical College and Hospital (SCBMCH) and the Sardar Vallabh Bhai Patel Postgraduate Institute of Paediatrics (SVPPGIP), Cuttack. Both institutions are tertiary care referral centers serving the entire population of Odisha. The study period extended from January 2023 to July 2024. All enrolled neonates were followed until six weeks postnatal age.

Study population

All neonates admitted to SCBMCH and SVPPGIP during the study period with a BW < 1,500 g and a GA < 34 weeks were included. Neonates with significant congenital anomalies, ocular malformations, or requiring ventilator support at admission were excluded.

Sample size calculation

The sample size was calculated based on reported ROP prevalence in Indian studies, ranging from 18% to 26%. Assuming a prevalence of 18%, a 95% confidence interval (CI), and a 5% margin of error, the required sample size was 236. Accounting for a 10% loss to follow-up, a total of 260 neonates were enrolled. During the study, 30 neonates were excluded due to ventilator requirement: six died, their parents withdrew 20, and four were found to have congenital anomalies. Ultimately, data from 200 preterm or VLBW neonates were analyzed.

Ethical considerations

Informed consent was obtained from parents after explaining the study objectives, procedures, and potential risks in their vernacular language. Institutional ethical committee approval was obtained before the commencement of the study.

Data collection

Detailed clinical history and potential risk factors were documented using a structured proforma. Neonatal weight was measured without clothing using an electronic weighing machine with 10 g precision. Serial daily weight monitoring was conducted during hospitalization and at follow-up visits until six weeks postnatal age. Average daily weight gain was calculated using the following formula: (weight at the time of examination − weight measured previously)/number of days between the two measurements.

Statistical analysis

Data were analyzed using SPSS version 30 (IBM Corp., Armonk, NY, USA). Continuous variables were expressed as mean ± standard deviation, while categorical variables were presented as frequencies and percentages. Univariate analysis was performed for relevant variables, and associations between categorical variables were assessed using Pearson’s chi-squared test or the Mann-Whitney test as appropriate. Risk factors for ROP were analyzed using binomial logistic regression to determine their association with disease occurrence. A p-value < 0.05 was considered statistically significant.

## Results

A total of 200 VLBW preterm infants were included in the study, comprising 110 (55.0%) male infants and 90 (45.0%) female infants. Severe ROP was identified in 76 (38.0%) infants. The incidence of severe ROP showed a significant inverse relationship with GA, occurring in 23 (92.0%) infants born at <28 weeks, 30 (66.7%) born at 28-30 weeks, 17 (39.5%) born at 30-32 weeks, and six (6.9%) born at 32-34 weeks (p < 0.001). BW was also significantly associated with severe ROP, which occurred in 51 (66.2%) infants weighing ≤1,100 g, 21 (46.7%) infants weighing 1,100-1,300 g, and four (5.1%) infants weighing >1,300 g (p < 0.001). Severe ROP occurred in 45 (78.9%) infants with anemia, 11 (73.3%) infants with bronchopulmonary dysplasia (BPD), and 32 (84.2%) infants with apnea, all of which were significantly associated with severe ROP (p < 0.05). In contrast, weight for GA, IVH, and sepsis were not significantly associated with severe ROP. Infants with severe ROP had significantly higher blood transfusion requirements (0.7 ± 0.9 vs. 0.1 ± 0.4) and a longer duration of oxygen therapy (14.5 ± 8.3 vs. 6.0 ± 5.4 days) than those without severe ROP (both p < 0.001) (Table 1).

**Table 1:** Association of clinical variables with severe retinopathy of prematurity (ROP) (N = 200) Data are presented as n (%) for categorical variables and as mean ± standard deviation (SD) with median (interquartile range (IQR)) for continuous variables. Pearson's chi-squared (χ²) test (or Fisher's exact test where appropriate) was used to compare categorical variables, with Cramer's V reported as the measure of effect size. Continuous variables were compared using the Mann-Whitney U test. A p-value of <0.05 was considered statistically significant. *Significant p-value. AGA: appropriate for gestational age; SGA: small for gestational age; LGA: large for gestational age; BPD: bronchopulmonary dysplasia; IVH: intraventricular hemorrhage

Variable	Category	Severe ROP		Total, n (%)	Chi square (df)	Effect size Cramer	p-value
		No, N (%)	Yes, N (%)				
Gestational age (GA) (weeks)	<28	2 (8.0)	23 (92.0)	25 (100)	66.6 (3)	0.577	<0.001*
	28-30	15 (33.3)	30 (66.7)	45 (100)			
	30-32	26 (60.5)	17 (39.5)	43 (100)			
	32-34	81 (93.1)	6 (6.9)	87 (100)			
Weight for GA	AGA	98 (59.4)	67 (40.6)	165 (100)	5.13 (2)	0.160	0.077*
	SGA	26 (76.5)	8 (23.5)	34 (100)			
	LGA	0 (0)	1 (100)	1 (100)			
Birth weight (g)	≤1,100	26 (21.0)	51 (67.1)	77 (38.5)	58.8 (2)	0.543	<0.001*
	1,100-1,300	24 (19.4)	21 (27.6)	45 (22.5)			
	>1,300	74 (59.7)	4 (5.3)	78 (39.0)			
Anemia	No	112 (78.3)	31 (21.7)	143 (71.5)	59.2 (1)	0.544	<0.001*
	Yes	12 (21.1)	45 (78.9)	57 (28.5)			
BPD	No	120 (64.9)	65 (35.1)	185 (92.5)	8.98 (1)	0.212	0.003*
	Yes	4 (26.7)	11 (73.3)	15 (7.5)			
IVH	No	101 (63.9)	57 (36.1)	158 (79.0)	1.18 (1)	0.077	0.277
	Yes	23 (54.8)	19 (45.2)	42 (21.0)			
Apnea	No	118 (72.8)	44 (27.2)	162 (81.0)	38.96 (1)	0.441	<0.001*
	Yes	6 (15.8)	32 (84.2)	38 (19.0)			
Sepsis	No	81 (66.9)	40 (33.1)	121 (60.5)	3.17 (1)	0.126	0.075
	Yes	43 (54.4)	36 (45.6)	79 (39.5)			
Blood transfusions	Mean ± SD	0.1 ± 0.4	0.7 ± 0.9	-	Z = -5.61	-	<0.001
	Median (IQR)	0 (0-0)	0 (0-1)	-			
Days of oxygen therapy	Mean ± SD	6.0 ± 5.4	14.5 ± 8.3	-	t = -8.24	-	<0.001
	Median (IQR)	5 (3-6)	14 (8-18)	-			

Regarding disease characteristics, Zone 1 ROP was observed in 43 (21.5%) infants, Zone 2 in 53 (26.5%) infants, and Zone 3 in 104 (52%) infants. With respect to staging, Stage 1 ROP was noted in 76 (38%) infants, Stage 2 in 85 (42.5%) infants, and Stage 3 in 39 (19.5%) infants; no cases of Stage 4 or Stage 5 ROP were identified. Plus disease was present in 76 (38%) infants. At final follow-up, 30 (15%) infants showed regressed ROP, 76 (38%) infants had regressing disease, and 94 (47%) infants had a mature retina. Among the 76 infants requiring treatment, 52 (68.4%) received intravitreal bevacizumab, while 24 (31.6%) underwent laser photocoagulation (Table 2).

**Table 2:** Final outcome of ROP ROP: retinopathy of prematurity

Variables	Classification	Total number, N (%)
Zone	Zone 1	43 (21.5%)
	Zone 2	53 (26.5%)
	Zone 3	104 (52%)
Stage	Stage 1	76 (38%)
	Stage 2	85 (42.5%)
	Stage 3	39 (19.5%)
	Stage 4	0 (0%)
	Stage 5	0 (0%)
Plus disease	No	124 (62%)
	Yes	76 (38%)
ROP final stage	Regressing	76 (38%)
	Regressed ROP	30 (15%)
	Mature retina	94 (94%)
Treatment (N = 76)	Avastin injection	52 (68.4%)
	Laser	24 (31.6%)

Postnatal weight gain was consistently lower in infants who developed severe ROP across all postnatal weeks. During the second week, infants without severe ROP gained 9.7 ± 2.1 g/day compared with 5.9 ± 3.7 g/day in those with severe ROP (p < 0.001). Similar differences were observed in the third week (15.2 ± 2.4 vs. 9.2 ± 4.2 g/day), fourth week (17.6 ± 3.1 vs. 10.4 ± 4.8 g/day), fifth week (19.3 ± 2.7 vs. 10.3 ± 3.9 g/day), and sixth week (19.5 ± 2.7 vs. 10.8 ± 4.4 g/day), with all comparisons reaching statistical significance (p < 0.001). Infants with severe ROP also required a significantly longer duration to regain BW (22.2 ± 5.9 days) compared to those without severe ROP (14.4 ± 1.7 days; p < 0.001) (Table 3).

**Table 3:** Comparison of average weight gain per day in different weeks between severe ROP A p-value less than 0.05 was considered significant. *Significant p-value. ROP: retinopathy of prematurity; IQR: interquartile range; SD: standard deviation

Average weight gain per day in different weeks	Severe ROP				Mann-Whitney U	p-value
	No (N = 124)		Yes (N = 76)			
	Mean ± SD	Median (IQR)	Mean ± SD	Median (IQR)		
2nd week	9.7 ± 2.1	9.7 (8.5 to10.6)	5.9 ± 3.7	4.8 (2.5-8.4)	-7.02	<0.001*
3rd week	15.2 ± 2.4	14.8 (13.7 to 15.8)	9.2 ± 4.2	8.5 (5.8-12.3)	-8.14	<0.001*
4th week	17.6 ± 3.1	17.4 (15.6 to 19.0)	10.4 ± 4.8	10.7 (6.5-12.5)	-8.73	<0.001*
5th week	19.3 ± 2.7	18.7 (17.5 to 20.5)	10.3 ± 3.9	9.0 (7.2-13.1)	-10.21	<0.001*
6th week	19.5 ± 2.7	19.5 (17.5 to 20.5)	10.8 ± 4.4	10.2 (8.3-12.8)	-9.56	<0.001*
Days for attaining birth weight	14.4 ± 1.7	14.0 (13.0 to 16.0)	22.2 ± 5.9	20.0 (19.0-24.0)	8.92	<0.001*

Binary logistic regression analysis identified GA, apnea, BW, and postnatal weight gain as independent predictors of severe ROP (Nagelkerke R² = 0.839). Compared with infants born at 32-34 weeks (reference category), those born at ≤28 weeks had an 81.64-fold higher odds of developing severe ROP (odds ratio (OR) = 81.639, 95% CI: 7.175-928.922, p < 0.001), those born at 28-30 weeks had an 8.93-fold higher odds (OR = 8.929, 95% CI: 1.313-60.710, p = 0.025), and those born at 30-32 weeks had a 17.32-fold higher odds (OR = 17.320, 95% CI: 2.442-122.842, p = 0.004). Infants with apnea had 34.59 times higher odds of severe ROP than those without apnea (OR = 34.594, 95% CI: 5.398-221.697, p < 0.001). Higher postnatal weight gain during the second and third weeks was independently associated with a lower risk of severe ROP. Specifically, each one-unit increase in average weight gain reduced the odds of severe ROP by 30.1% during the second week (OR = 0.699, 95% CI: 0.576-0.847, p < 0.001) and by 44.4% during the third week (OR = 0.556, 95% CI: 0.435-0.711, p < 0.001). Additionally, infants in the lowest BW category (≤1,100 g) had 11.37 times higher odds of developing severe ROP than the reference BW group (OR = 11.371, 95% CI: 1.580-81.863, p = 0.016) (Table 4).

**Table 4:** Binary logistic regression analysis of factors associated with severe retinopathy of prematurity Variables included in the first step of the binary logistic regression model were gestational age (GA), bronchopulmonary dysplasia (BPD), apnea, number of blood transfusions, duration of oxygen therapy, average weight gain during the second and third postnatal weeks, anemia, and birth weight category. Gestational age was divided into four groups: ≤28 weeks, 28-30 weeks, 30-32 weeks, and 32-34 weeks, with the latter serving as the reference category. CI: confidence interval

Variables	B	S.E.	Wald	df	Sig.	Odds ratio	95% CI for EXP (B)		Nagelkerke R square
							Lower	Upper	0.839
GA (Ref)	-	-	13.554	3	0.004	-	-	-	
GA (1)	4.402	1.241	12.59	1	<0.001	81.639	7.175	928.922	
GA (2)	2.189	0.978	5.012	1	0.025	8.929	1.313	60.710	
GA (3)	2.852	1.000	8.141	1	0.004	17.320	2.442	122.842	
Apnea (1)	3.544	0.948	13.98	1	<0.001	34.594	5.398	221.697	
2nd week	-0.359	0.098	13.292	1	<0.001	0.699	0.576	0.847	
3rd week	-0.587	0.125	22.037	1	<0.001	0.556	0.435	0.711	
Birth weight group (1)	2.431	1.007	5.827	1	0.016	11.371	1.580	81.863	
Constant	6.987	2.03	11.85	1	<0.001	1,082.648			

## Discussion

This study provides important insights into the multifactorial nature of ROP, focusing on postnatal weight gain and its relationship to disease severity in preterm infants. ROP is a disorder of the developing retina, influenced by a combination of prenatal and postnatal factors. Evidence from the Indian subcontinent regarding the role of postnatal weight gain in severe ROP is limited, highlighting the relevance of the present study. The study aimed to assess the effect of postnatal weight gain on ROP progression and severity, alongside other postnatal risk factors.

In our cohort, there was a statistically significant difference in weight gain during the first six weeks of life (p < 0.0001) between neonates who developed severe ROP and those who did not. Binary logistic regression analysis, controlling for potential confounders, revealed an inverse relationship between average weight gain in the second and third weeks and the risk of severe ROP. This indicates that higher weight gain during these early weeks is associated with a lower risk of severe disease.

These results are consistent with findings from other studies. Chaves-Samaniego et al. [13] reported that neonates who did not require treatment for ROP had higher mean weight gain during the first four weeks of life (12.75 ± 5.99 g/day) compared to those who needed treatment (9.50 ± 5.45 g/day, p < 0.001). Similarly, Sethi et al. [14] observed significant differences in weight gain between preterm infants with and without treatable ROP, with mean gains of 33.12 and 15.31 g/day, respectively (p = 0.001). Doshi et al. [15] reported that infants with severe ROP had a mean weight gain of 11.56 ± 7.77 g/day, compared to 18.21 ± 10.84 g/day in the overall cohort. Together, these studies support the conclusion that early postnatal weight gain is a critical factor influencing the development of ROP.

While low GA and BW remain primary risk factors for ROP, other postnatal factors such as prolonged oxygen therapy, BPD, anemia, and repeated blood transfusions also contribute to disease severity. Monitoring postnatal weight gain provides an indirect measure of overall health, nutritional status, and the effectiveness of neonatal care, all of which can influence ROP progression. In this study, inadequate weight gain during the second and third weeks of life emerged as a significant risk factor for severe ROP.

Despite these findings, the multifactorial nature of ROP necessitates further research. Larger, multicenter studies with careful control of confounding variables and standardized follow-up protocols are needed to establish a definitive cause-and-effect relationship between early postnatal weight gain and ROP severity. Nevertheless, this study emphasizes the importance of monitoring weight gain in preterm infants to support early identification and preventive strategies for ROP.

Limitations

This was a single-center study, which limits the diversity of the study population. Additionally, antenatal risk factors for ROP were not assessed.

## Conclusions

This study demonstrates that ROP is strongly influenced by perinatal and early postnatal factors, particularly GA, BW, early postnatal weight gain, and apnea. Optimizing early postnatal growth and ensuring close surveillance of high-risk infants may help reduce the risk of progression to severe ROP. These findings emphasize the importance of early identification and targeted interventions in vulnerable preterm infants to prevent disease progression and improve clinical outcomes.
